# Kaempferol Improves Cardiolipin and ATP in Hepatic Cells: A Cellular Model Perspective in the Context of Metabolic Dysfunction-Associated Steatotic Liver Disease

**DOI:** 10.3390/nu16040508

**Published:** 2024-02-11

**Authors:** Akiko Sakurai, Toshihiro Sakurai, Hsin-Jung Ho, Hitoshi Chiba, Shu-Ping Hui

**Affiliations:** 1 Faculty of Health Sciences, Hokkaido University, Sapporo 060-0812, Japan; sakurai.akiko.p6@elms.hokudai.ac.jp (A.S.); hsinjung@hs.hokudai.ac.jp (H.-J.H.); 2 Department of Nutrition, Sapporo University of Health Sciences, Sapporo 007-0894, Japan; chiba-h@sapporo-hokeniryou-u.ac.jp

**Keywords:** metabolic dysfunction-associated steatotic liver disease, metabolic dysfunction-associated steatohepatitis, liquid chromatography/mass spectrometry, lipidomics, monolysocardiolipin, ATP

## Abstract

Targeting mitochondrial function is a promising approach to prevent metabolic dysfunction-associated steatotic liver disease (MASLD). Cardiolipin (CL) is a unique lipid comprising four fatty acyl chains localized in the mitochondrial inner membrane. CL is a crucial phospholipid in mitochondrial function, and MASLD exhibits CL-related anomalies. Kaempferol (KMP), a natural flavonoid, has hepatoprotective and mitochondrial function-improving effects; however, its influence on CL metabolism in fatty liver conditions is unknown. In this study, we investigated the effects of KMP on mitochondrial function, focusing on CL metabolism in a fatty liver cell model (linoleic-acid-loaded C3A cell). KMP promoted mitochondrial respiratory functions such as ATP production, basal respiration, and proton leak. KMP also increased the gene expression levels of *CPT1A* and *PPARGC1A*, which are involved in mitochondrial β-oxidation. Comprehensive quantification of CL species and related molecules via liquid chromatography/mass spectrometry showed that KMP increased not only total CL content but also CL72:8, which strongly favors ATP production. Furthermore, KMP improved the monolysocardiolipin (MLCL)/CL ratio, an indicator of mitochondrial function. Our results suggest that KMP promotes energy production in a fatty liver cell model, associated with improvement in mitochondrial CL profile, and can serve as a potential nutrition factor in preventing MASLD.

## 1. Introduction

Nonalcoholic fatty liver disease (NAFLD) is the most common chronic liver disease and affects approximately 25% of the global adult population [1]. NAFLD is caused by the accumulation of excess lipids in the liver, leading to simple steatosis that can progress to nonalcoholic steatohepatitis (NASH), liver cirrhosis, and hepatocellular carcinoma due to oxidative stress [2]. The prevalence of NAFLD is rapidly increasing alongside the global increase in the incidence of metabolic syndrome, diabetes, and obesity [3]. NAFLD is associated with an increased risk of cardiovascular disease, complications of type 2 diabetes, and hepatocellular carcinoma [4]. Therefore, elucidating the mechanisms underlying the pathogenesis of NAFLD is important for managing NAFLD and its comorbidities. Recently, NAFLD/NASH has been described as a metabolic disease that includes dyslipidemia, hyperglycemia, and hypertension and has been reconceptualized as metabolic dysfunction-associated fatty liver disease (MAFLD), metabolic dysfunction-associated steatotic liver disease (MASLD), or metabolic dysfunction-associated steatohepatitis (MASH) [5]. A recent study reported that 99.5% of patients with NAFLD met MASLD criteria [6]. Overall, MASLD/MASH must be studied for its mechanism and treatment.

Currently, NAFLD/MASLD lacks approved drug treatments. Mitochondrial dysfunction may be associated with fat accumulation and liver dysfunction. NAFLD causes both mitochondrial structural and functional abnormalities, such as decreased β-oxidation, excessive production of reactive oxygen species and lipid hydroperoxides, and abnormalities in the electron transport chain [7]. Thus, the development of new prevention and treatment methods for NAFLD/MASLD that target mitochondrial function is gaining increasing attention.

Cardiolipin (CL) is a phospholipid specific to mitochondria and is localized in the inner mitochondrial membrane. CL plays an important role in mitochondrial structure and function during adenosine triphosphate (ATP) production in the inner mitochondrial membrane [8]. Mitochondria have the following unique pathways for CL synthesis: Nascent CL is synthesized in the mitochondria [9] and then deacylated by a patatin-like phospholipase domain-containing protein 8, resulting in the formation of monolysocardiolipin (MLCL) [10]. Subsequently, mature CL is synthesized by reacylation with tafazzin (TAZ) and lysocardiolipin acyltransferase 1. This process is called CL remodeling. TAZ selectively binds linoleic acid (LA) during CL formation [11]. CL 72:8 is bound to four linoleic acyl groups and is considered functional or mature CL [12]. TAZ-deficient patients have a marked decrease in CL 72:8 and accumulation of MLCL, accompanied by a marked decline in movement function [13]. Therefore, CL72:8 significantly influences energy metabolism and is related to movement functions.

Kaempferol (KMP; 3,5,7-trihydroxy-2-(4-hydroxyphenyl)-4H-chromen-4-one) is a flavonoid aglycone found in foods such as tea leaves, spinach, kale, and blueberries [14]. In vitro and in vivo studies have shown KMP to have hepatoprotective [15], anti-fatty liver [16], antioxidant [17], and anti-inflammatory effects [18]. Moreover, mitochondrial function-improving effects of KMP have been reported; KMP increased ATP production in mouse myotube differentiated from C2C12 under hypoxic conditions [19]. However, the mitochondrial function-improving effect of KMP in fatty liver has not been investigated. In particular, the effects of KMP on improving CL metabolism remain unknown.

Therefore, to explore the potential application of KMP in NAFLD/MASLD, we aimed to assess whether KMP improves mitochondrial function, especially CL metabolism, in cultured liver cells.

## 2. Materials and Methods

### 2.1. Cell Culture

Human liver-derived C3A cells were purchased from the American Type Culture Collection. The C3A cells were cultured at 37 °C and 5% CO_2_ using minimum essential medium (MEM, Thermo Fisher Scientific, Waltham, MA, USA) supplemented with GlutaMAX^TM^ (Thermo Fisher Scientific) and 10% fetal bovine serum (FBS, Thermo Fisher Scientific) and 1% penicillin-streptomycin-neomycin (PSN, Thermo Fisher Scientific).

### 2.2. Cell Viability Test

To confirm the cytotoxic concentration of KMP, a cell viability test was performed using a water-soluble tetrazolium salt WST-1 assay (*n* = 6 for each group; Table S1). Briefly, 1.0 × 10^5^ C3A cells/mL (MEM containing 10% FBS) were seeded in a 96-well plate at 100 µL/well and precultured for 24 h at 37 °C and 5% CO_2_. Subsequently, the cell culture medium was replaced with KMP (100 µL/well) (Cayman Chemical, Ann Arbor, MI, USA) solution, and C3A was stimulated for 24 h at 37 °C and 5% CO_2_. The concentration of each KMP solution was adjusted using 99.5% ethanol to achieve a final concentration ranging from 0 to 100 µM, as previously described [17]. It was mixed in supplemented clear MEM (no FBS, no glutamine, no phenol red; Thermo Fisher Scientific) (final ethanol concentration: 0.5%). The WST-1 reagent comprised a 9:1 mixture of WST-1 (FUJIFILM Wako Pure Chemical Industries, Ltd., Osaka, Japan) and 1-methoxy-phenazine methosulfate (FUJIFILM Wako Pure Chemical Industries, Ltd.) and was added 22 h after stimulation at 10 µL/well. After incubation for 2 h, the absorbance at 450 nm was measured using a plate reader (xMark™ Microplate Spectrophotometer, Bio-Rad Laboratories, Inc., Hercules, CA, USA). Each KMP solution was used as a blank. The cell survival rate was expressed as a relative value, with the control set at 100%.

### 2.3. Mitochondrial Functional Analysis

To assess the energy metabolic state of the cells, oxygen consumption rate (OCR) was measured using a Seahorse XFp extracellular analyzer (Agilent Technologies, Santa Clara, CA, USA; Table S1). Briefly, C3A cells were precultured for 24 h under 5% CO_2_ conditions. Thereafter, 150 µL of the cell culture medium was replaced with clear MEM (0% FBS) containing ethanol (final ethanol concentration: 0.5%) (control group) or clear MEM (0% FBS) containing 10 µM of KMP (0.5% ethanol at final concentration) and incubated at 37 °C and 5% CO_2_ for 24 h. Furthermore, 150 µL of the medium was replaced with 130 µL of Seahorse XF DMEM (Agilent Technologies), containing 10 mM of glucose (final concentration, Nacalai Tesque, Kyoto, Japan), 1 mM of pyruvate (Thermo Fisher Scientific), and 2 mM of GlutaMAX^TM^ (Thermo Fisher Scientific) at pH 7.4, and incubated for 1 h at 37 °C without CO_2_. Subsequently, the measurements were performed at 37 °C, during which a mixture of oligomycin (final concentration: 4 µM), carbonyl cyanide 4-(trifluoromethoxy) phenylhydrazone (final concentration: 1 µM), and antimycin A and rotenone (final concentration: 0.5 µM), contained in the Mito Stress Test Kit (Agilent Technologies), was used. Then, 20 µL each of the reagents above was injected into each well stepwise. The OCR was analyzed using the Wave 2.6.1 software provided by Agilent Technologies. Six parameters, including basal respiration, ATP production, proton leak, maximal respiration, reserve respiration, and non-mitochondrial respiration, were calculated from the OCR area under the curve values.

### 2.4. Real-Time Polymerase Chain Reaction (PCR)

Real-time PCR was performed to analyze the expression of each target gene: carnitine palmitoyltransferase 1A (*CPT1A*), which is related to β-oxidation; and sirtuin 3 (*SIRT3*), forkhead box class O3a (*FOXO3A*), peroxisome proliferator-activated receptor gamma coactivator 1α (*PPARGC1A*), and mitochondrial transcription factor A (*TFAM*), which are related to mitochondrial biogenesis (Table S1). Briefly, C3A cell suspension (10% FBS) containing 2.0 × 10^5^ cells/mL was seeded in a 24-well plate at 1 mL/well and precultured for 24 h at 37 °C and 5% CO_2_. Thereafter, the cell culture medium was replaced with KMP solution (1 mL/well), and the cells were stimulated for 6 h at 37 °C and 5% CO_2_. The concentration of KMP was adjusted using ethanol to achieve final concentrations of 0, 1, and 10 μM; it was mixed in clear MEM (0% FBS) (final ethanol concentration at 0.5%). Cells were harvested, and RNA was extracted using the PureLink™ RNA Mini Kit (Thermo Fisher Scientific) according to the manufacturer’s instructions. The concentration and integrity of the isolated RNA were determined at OD 260/280 using a NanoDrop spectrophotometer (Thermo Fisher Scientific). RNA was converted to complementary DNA (cDNA) using ReverTra Ace^®^ qPCR RT Master Mix with gDNA Remover (Toyobo, Co., Ltd., Osaka, Japan). Real-time PCR was performed using the CFX 96 Real-Time PCR Detection System (Bio-Rad Laboratories Inc.) and Thunderbird^®^ SYBR qPCR Mix (Toyobo) according to the manufacturer’s instructions. Each experiment was repeated thrice. The primer sequences are listed in Table S3 [20,21,22,23,24,25]. The PCR reaction was performed under the following conditions: initial denaturation at 95 °C for 60 s, followed by 40 cycles of denaturation at 95 °C for 15 s, and extension at 60 °C for 30 s. The expression levels were determined and normalized to those of *β-actin*. The data were expressed as values relative to the expression level of the 0 µM KMP group.

### 2.5. Mitochondrial DNA (mtDNA) Copy Number

Cells were stimulated for 24 h under the same conditions as those used in real-time PCR experiments. After removal of the cell culture medium, the cells were detached using TrypLE (Thermo Fisher Scientific), and clear MEM containing 10% FBS was added. After the cells were collected, they were centrifuged (himac CE15R, Hitachi Koki Co., Ltd., Tokyo, Japan, 15,000× *g*, 4 °C, 1 min). The supernatant was discarded, and samples were collected for mtDNA copy number measurement.

For DNA extraction, the cells were collected using a DNA isolation kit for mammalian blood (Roche, Basel, Switzerland) according to the manufacturer’s instructions. DNA was extracted from the cells and subjected to real-time PCR. The primer sequences are listed in Table S3. The expression of NADH-ubiquinone oxidoreductase chain 1 (*ND1*), used as a marker of mtDNA copy number, was normalized to that of glyceraldehyde-3-phosphate dehydrogenase (*GAPDH*).

### 2.6. Pretreatment of LA–Bovine Serum Albumin (BSA) Conjugate

CL72:8 (mostly 18:2 × 4) is a molecular species that should be considered for the evaluation of functional CL; however, its concentration is low in cultured cells. Therefore, to compensate for this low value, LA was added to C3A cells to promote the formation of lipid droplets and increase CL72:8 concentration in this fatty liver model. First, a stock solution of LA (Tokyo Chemical Industry Co., Ltd., Tokyo, Japan) (1 M) was prepared by dissolving LA in 99.5% ethanol. Next, to bind LA in ethanol with fatty-acid-free BSA in phosphate-buffered saline (PBS), 1 M of LA stock solution (1 M) and 30% BSA–PBS were mixed at a ratio of 3:500 (vol:vol) and incubated at 37 °C for 1 h, with reference to previous reports [26,27]. Finally, the LA–BSA conjugate solution (final concentration: 6 mM) was stored at −80 °C. A solution containing equal parts BSA and ethanol was prepared instead of using 1 M of LA, serving as 0 µM of LA or a dilution of the LA–BSA conjugate solution.

### 2.7. CL and MLCL Analysis via Liquid Chromatography/Mass Spectrometry (LC/MS)

The effects of KMP on CL metabolism were determined by assessing CL and MLCL in a fatty liver cell model with or without KMP treatment using LC/MS (*n* = 5–6 for each group; Table S2), as previously reported [28]. In the process, 2.0 × 10^5^ C3A cells/mL (clear MEM containing 10% FBS) were seeded in a 24-well plate at 1 mL/well and precultured for 24 h at 37 °C and 5% CO_2_. Thereafter, the cell culture medium was replaced with a mixture of the LA–BSA conjugate and KMP at a volume of 1 mL/well. Each LA–BSA complex and KMP mixture was adjusted to a final concentration of 0.8 mM for LA–BSA complex and 0, 1, or 10 µM for KMP and mixed in clear MEM containing 0% FBS (final ethanol concentration: 0.5%). The cells were incubated for 24 h at 37 °C and 5% CO_2_. After removing the culture medium, cells were detached using 300 µL/well of TrypLE, and then 100 µL/well of clear MEM containing 10% FBS was added. The cells were collected into 1.5 mL tubes and were centrifuged (himac CE15R, 15,000× *g*, 4 °C, 5 min). The supernatant was discarded, and 500 µL of PBS was added and centrifuged again (15,000× *g*, 4 °C, 5 min). The supernatant was discarded, and the cells were washed with 500 µL of PBS. After mixing, 50 µL of this cell suspension was collected for measurement of protein concentration. The samples were stored at −80 °C. The remaining 450 µL of cell suspension was centrifuged (15,000× *g*, 4 °C, 5 min) again, and the supernatant was discarded. The cell samples were stored at −80 °C until lipid extraction.

Lipid extraction from cells was performed as described by Folch et al. (*n* = 6 per group) [29]. A liquid chromatography–tandem mass spectrometer (LTQ Orbitrap XL, Thermo Fisher Scientific) was used for the simultaneous analysis of lipids (CL and MLCL) in cultured liver C3A cells, as described previously [30]. We used the Shimadzu liquid chromatograph system (DGU-20A_3_, LC-20AD, SIL-20A, CTO-20A, Shimadzu, Kyoto, Japan) equipped with an Atlantis T3 column (3 μm, 2.1 mm × 150 mm; Waters, Milford, MA, USA) for the separation of lipids. Peak areas were calculated using Xcalibur 2.2 (Thermo Fisher Scientific). The CL and MLCL values were expressed by correcting the peak area with the protein concentration. Cellular protein concentration was determined using the Pierce™ BCA Protein Assay Kits (Thermo Fisher Scientific) according to the manufacturer’s instructions.

### 2.8. Statistical Analysis

All statistical analyses were performed using GraphPad Prism V7.0/10.1.2 software. After performing a rejection test by identifying the outliers, cell viability was evaluated using a one-way analysis of variance (ANOVA), followed by Dunnett’s multiple comparison test. Mitochondrial function was evaluated using an unpaired *t*-test. For other analyses, multiple comparison tests were performed using one-way ANOVA with Tukey’s multiple comparison test. The level of significance was set at 5%. All values are expressed as the mean ± standard deviation (SD).

## 3. Results

### 3.1. Cell Viability

**Figure 1:**
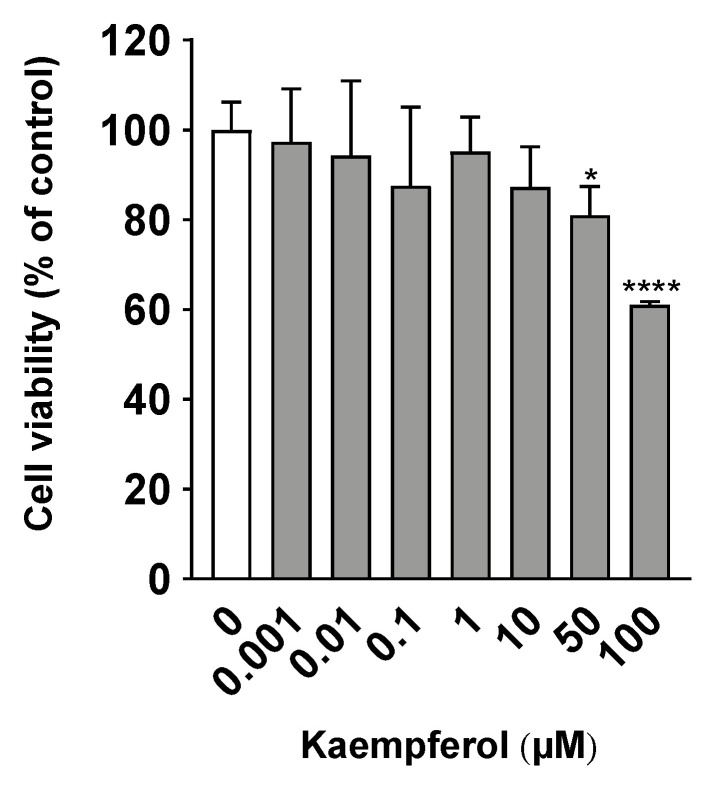
Effects of kaempferol on cell viability in C3A cells. Cell viability was measured using the WST-1 assay. Values are presented as means ± standard deviations (SDs) (*n* = 6 per group). * *p* < 0.05, **** *p* < 0.0001 vs. 0 µM of kaempferol using one-way analysis of variance (ANOVA) with Dunnett’s multiple comparisons test.

To determine the optimal concentration of KMP for application to C3A cells, a cell viability test was performed. Treatment with less than 50 µM of KMP after 24 h of incubation did not reduce cell viability (Figure 1). However, a decrease in the viability of C3A cells was observed at KMP concentrations of 50 µM and higher (*p* < 0.05, 0.0001). Similarly, a previous report showed that the viability of a hepatocellular carcinoma cell line, HepG2, after a 48 h culture in the presence of KMP showed no change at a concentration of 10 µM and lower [17]. Therefore, in this study, we adopted 10 µM as the supplementation concentration of KMP.

### 3.2. Mitochondrial Function

**Figure 2:**
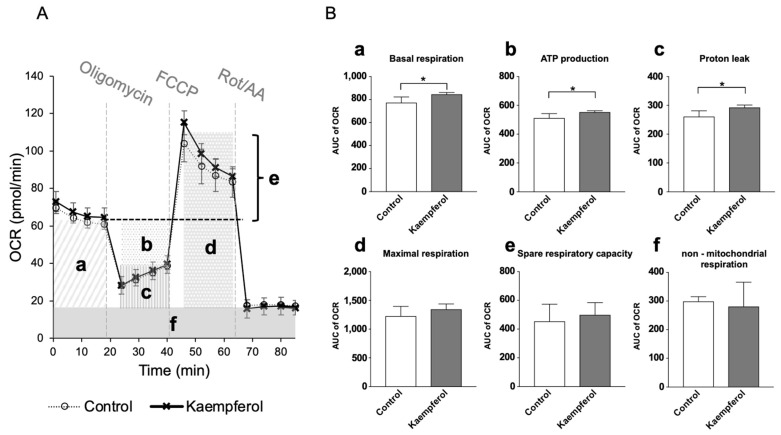
Effects of kaempferol on mitochondrial function in C3A cells. (**A**) Mitochondrial respiratory profile. Oxygen consumption rate (OCR) was measured over time (pmol/min) using an extracellular flux analyzer. Each reagent of the Mito Stress Test Kit (oligomycin, carbonyl cyanide 4-(trifluoromethoxy) phenylhydrazone [FCCP], or rotenone and antimycin A [Rot/AA]) was injected into each well stepwise. (**B**) The parameters were calculated according to each OCR value on mitochondrial oxidative phosphorylation, including (**a**) basal respiration, (**b**) ATP production, (**c**) proton leak, (**d**) maximal respiration, (**e**) spare respiration capacity, and (**f**) non-mitochondrial respiration. Values are presented as means ± standard deviations (SDs) (*n* = 4–5 per group). * *p* < 0.05, unpaired *t*-test.

Extracellular analysis was performed in duplicate to investigate the effects of KMP on mitochondrial function. Basal respiration, ATP production, and proton leak were significantly higher in the KMP group than in the control group (*p* < 0.05) (Figure 2). There were no significant differences in maximal respiration, spare respiratory capacity, or non-mitochondrial respiration.

### 3.3. Gene Expression Analysis

**Figure 3:**
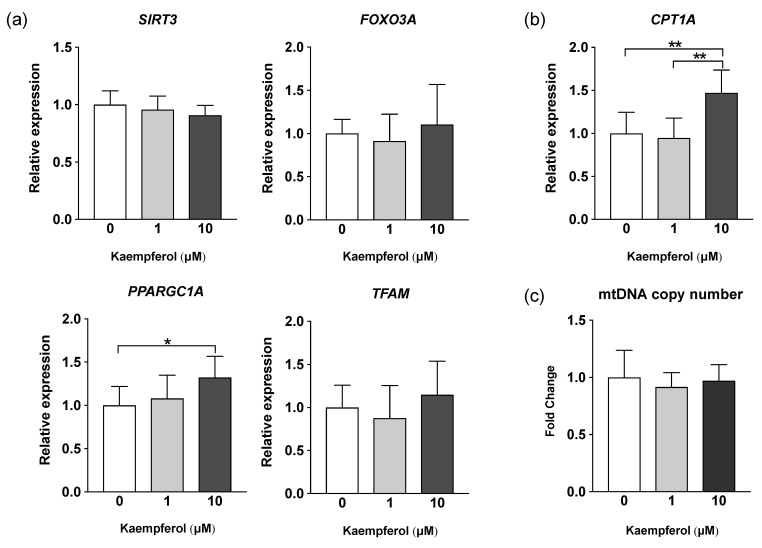
Effect of kaempferol on the expression of mitochondrial metabolism-related genes in C3A cells. (**a**) mRNA expression levels of mitochondrial biogenesis-related genes determined by real-time PCR. *β-actin* was used as a housekeeping control for each target gene. (**b**) mRNA expression levels of β-oxidation-related genes determined by real-time PCR. *β-actin* was used as a housekeeping control. (**c**) Mitochondrial DNA copy number determined by real-time PCR. *GAPDH* expression level was used as a control for nuclear DNA. All values are indicated as the relative expression compared with control group values, which were set to 1.0 (mean ± standard deviation [SD], *n* = 8 per group). * *p* < 0.05, ** *p* < 0.01, one-way analysis of variance (ANOVA) with Tukey’s multiple comparisons test.

Real-time PCR was performed to determine the expression of genes induced by KMP. KMP increased the gene expression of *CPT1A*, which encodes an enzyme related to β-oxidation, in a concentration-dependent manner (Figure 3). *CPT1A* expression was significantly higher in the 10 µM KMP group than in the 0 and 1 µM KMP groups (*p* < 0.01). Furthermore, the gene expression level of *PPARGC1A*, which is related to mitochondrial biogenesis and fatty acid oxidation, was significantly higher in the 10 µM KMP group than in the 0 µM KMP group (*p* < 0.05). No increase in the expression of any other mitochondrial biogenesis-related factors (*SIRT3*, *FOXO3A*, and *TFAM*) was found. Additionally, there was no significant difference in mitochondrial DNA copy number between the groups.

### 3.4. Profiles of CL Molecular Species

**Figure 4:**
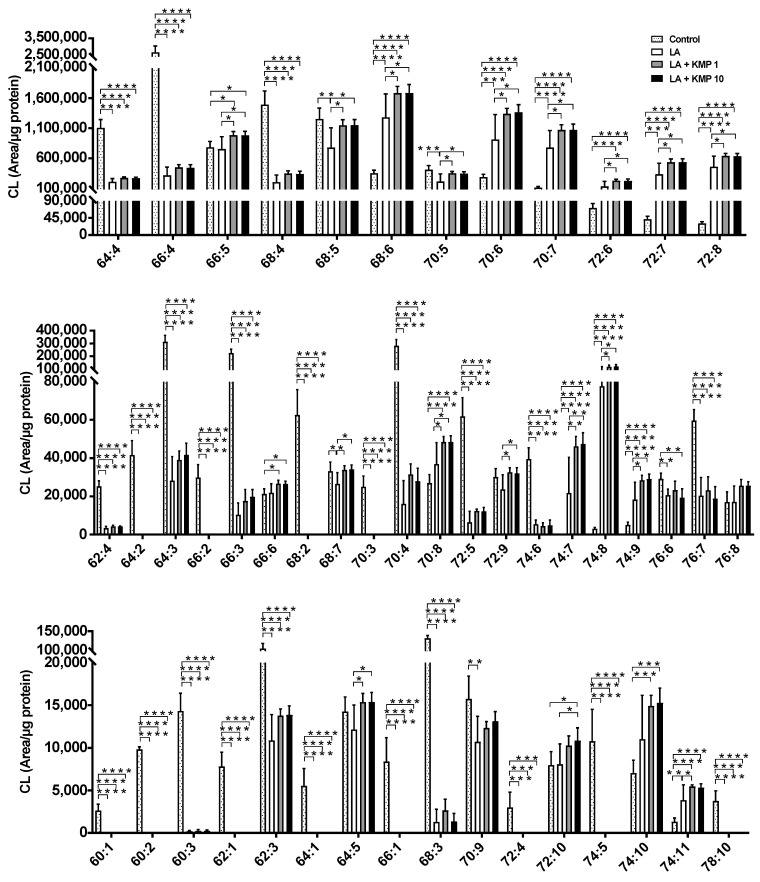
Efficacy of kaempferol in improving cardiolipin (CL) profiles in the linoleic acid (LA)-loaded fatty liver cell model. * *p* < 0.05, ** *p* < 0.01, *** *p* < 0.001, **** *p* < 0.0001, one-way analysis of variance (ANOVA) with Tukey’s multiple comparisons test.

**Figure 5:**
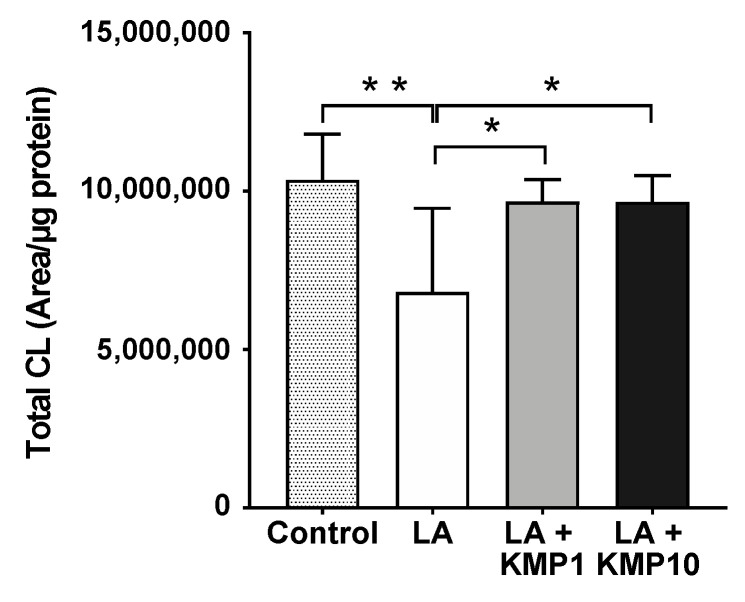
Efficacy of kaempferol in improving total CL level. Total CL was determined as the sum of the levels of all CL species determined in this study. * *p* < 0.05, ** *p* < 0.01, one-way analysis of variance (ANOVA) with Tukey’s multiple comparisons test.

Forty-eight CL species were detected in C3A cells of the fatty liver model (Figure 4). As expected, the CL72:8 levels were markedly increased by LA supplementation. Furthermore, CL72:8 levels were elevated in both the LA + KMP1 and LA + KMP10 groups compared to the LA group. The levels of CL molecular species with 5–9 double bonds were significantly increased in the LA + KMP1 and LA + KMP10 groups compared to the LA group. Total CL level was also significantly increased in the LA + KMP1 and LA + KMP10 groups compared to the LA group (Figure 5).

**Figure 6:**
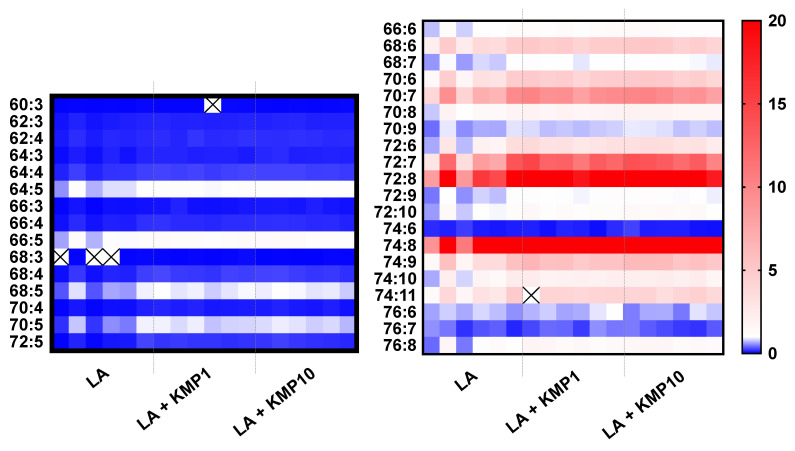
Heat map of cardiolipin (CL) species in the linoleic acid (LA)-loaded fatty liver cell model supplemented with or without KMP. With respect to color intensities, the mean values of each CL species in the control group are expressed as 1.0. Individual results for the samples are represented (*n* = 6 for each group). The cross mark means undetected CL species.

As shown in the heat map (Figure 6), the total levels of all CL species with five or fewer double bonds were significantly lower in the LA-treated groups than in the control group. Furthermore, the levels of more than half of the CL molecular species with six or more double bonds were significantly higher in the LA-treated groups than in the control group. Moreover, the ratio between each LA treatment group and the control group tended to be particularly high for CL molecular species with seven or eight double bonds. Notably, the cytoprotective effect of LA + KMP10 was observed in a cytotoxicity test by measuring the lactate dehydrogenase levels in the cell culture supernatant after stimulation (Figure S1).

### 3.5. Profiles of MLCL Species

**Figure 7:**
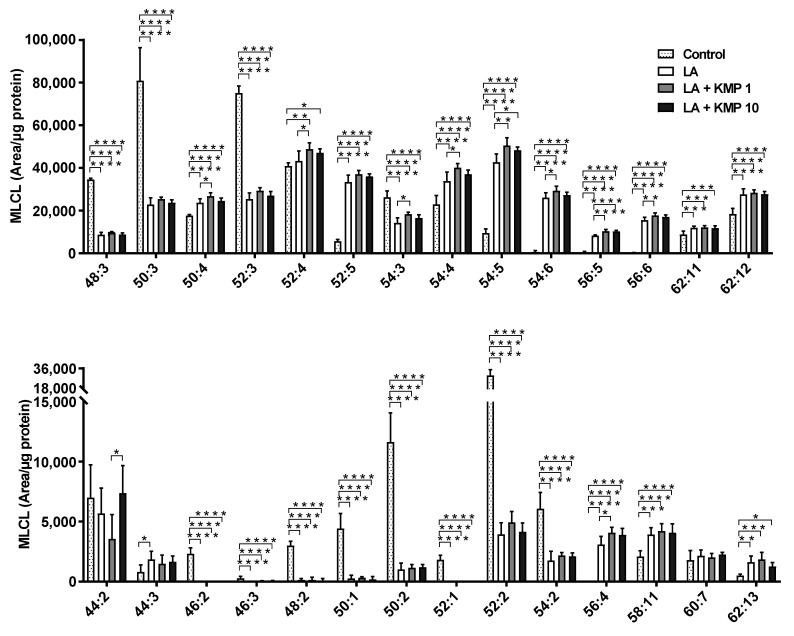
Efficacy of kaempferol in improving monolysocardiolipin (MLCL) profiles in the linoleic acid (LA)-loaded fatty liver cell model. * *p* < 0.05, ** *p* < 0.01, *** *p* < 0.001, **** *p* < 0.0001, one-way analysis of variance (ANOVA) with Tukey’s multiple comparisons test.

**Figure 8:**
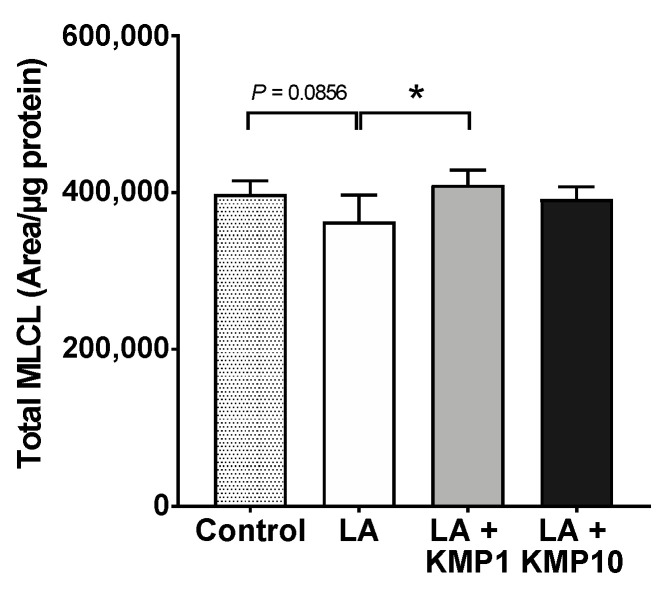
Efficacy of kaempferol in improving total MLCL levels. Total MLCL was determined as the sum of the levels of all MLCL species determined in this study. * *p* < 0.05, one-way analysis of variance (ANOVA) with Tukey’s multiple comparisons test.

Twenty-eight MLCL species were detected in C3A cells of the fatty liver cell model (Figure 7). The levels of most MLCL species with four or more double bonds were significantly higher in the LA-treated group than in the control group. By contrast, the levels of most MLCL species with three or fewer double bonds were significantly lower in the LA-treated group than in the control group. Finally, the levels of most MLCL species with 4–6 double bonds were significantly higher in the LA + KMP1 group than in the LA group. Total MLCL levels were also significantly higher in the LA + KMP1 group than in the LA group (Figure 8).

### 3.6. Total MLCL/Total CL Ratio 

**Figure 9:**
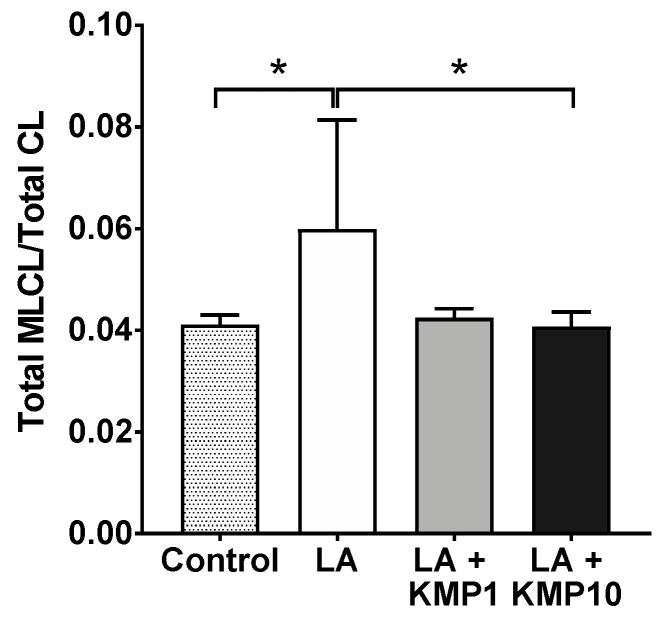
Efficacy of kaempferol in improving the balance of the Total MLCL/Total CL ratio in the LA-loaded fatty liver cell model. * *p* < 0.05, one-way analysis of variance (ANOVA) with Tukey’s multiple comparisons test.

To estimate the balance between CL and MLCL, the Total MLCL/Total CL ratio was determined as the sum of the levels in each species. The ratio was higher in the LA-alone supplementation group than in the control group. However, the ratio was significantly lower in the LA + KMP10 group than in the LA-alone supplementation group, comparable to the levels observed in the control group (Figure 9). Furthermore, the Total MLCL/Total CL ratio tended to be lower in the LA + KMP1 group than in the LA-alone supplementation group.

## 4. Discussion

**Figure 10:**
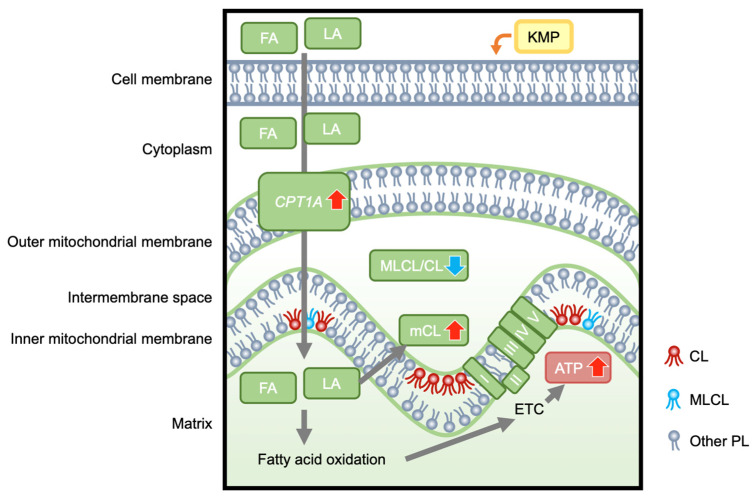
The hypothetical mechanism by which kaempferol enhances hepatocellular mitochondrial energetic metabolism, which is associated with improved cardiolipin profiles. KMP, kaempferol; FA, fatty acids; LA, linoleic acid; CPT1A, carnitine palmitoyltransferase 1A; MLCL, monolysocardiolipin; CL, cardiolipin; MLCL/CL, total MLCL/total CL ratio; mCL, mature CL; I, II, III, IV, V, Complex I-V; ETC, electron transport chain; ATP, adenosine triphosphate; PL, phospholipids.

In this study, we investigated the effects of KMP on mitochondrial function, focusing on CL metabolism in a fatty liver cell model (linoleic-acid-loaded C3A cell; Figure 10). Extracellular flux analysis revealed that KMP enhanced mitochondrial respiratory functions, including ATP production, in C3A cells. To the best of our knowledge, this is the first report demonstrating the improvement in mitochondrial respiratory function by KMP. Our findings suggest that KMP may enhance mitochondrial energy metabolism through a pathway that promotes the uptake of fatty acids into the mitochondria or a pathway that enhances mitochondrial biogenesis. CPT1A is localized in the outer mitochondrial membrane and plays a role in transporting long-chain fatty acids, such as LA, to the mitochondria [31]. CPT1A is the rate-limiting enzyme for β-oxidation of long-chain fatty acids, and activation of β-oxidation contributes to ATP production [32]. The overexpression of CPT1 has been shown to enhance hepatic mitochondrial fatty acid oxidation in mice [33]. KMP increased the protein expression of *CPT1A* in parallel with its gene expression in HepG2 cells [34]. KMP has also been shown to induce the protein expression of PPARGC1A in oleic-acid-induced HepG2 cells [35]. Furthermore, PPARGC1A overexpression in rats leads to fatty acid oxidation in the liver [36]. Thus, in this study, the increased expression of *CPT1A* and *PPARGC1A* induced by KMP may have contributed to increased fatty acid oxidation. By contrast, there were no changes in the expression of genes related to mitochondrial biogenesis or mtDNA. Therefore, the enhanced mitochondrial function by KMP in this study may be associated with the activation of β-oxidation based on the increase in the expression level of *CPT1A* rather than increased mitochondrial biogenesis.

CL is a lipid necessary for mitochondrial energy metabolism [8]. CL72:8, which contains four LA molecules, is the mature and functional form, and CL72:8 is the major component of hepatocytes in vivo [37]. In cultured C3A cells, the concentration of CL72:8 was only approximately 0.30% of the total concentration. Therefore, we developed a fatty liver model using LA. We observed the concentration-dependent formation of several lipid droplets at LA concentrations of 0–1 mM, without any change in the cytotoxicity rate in C3A cells. Sufficient lipid droplets were observed at 0.8 mM. This result is consistent with that reported by Tie et al., who observed several lipid droplets without any change in the viability of HepG2 cells after 24 h of culture in the presence of oleic acid (<1 mM) [17]. Therefore, in subsequent experiments, we used 0.8 mM of LA to generate a fatty liver cell model. In the preliminary stage of the study, mass spectrometry confirmed that the CL72:8 level increased with the addition of LA. Therefore, using LA instead of palmitic acid or oleic acid, which are often used to create fatty liver models, we not only achieved lipid droplets but also raised the baseline CL72:8 level.

CL72:8 was formed by supplying LA to the fatty liver cell model; however, LA supplementation decreased the total CL level. The present study demonstrated that the addition of KMP increased not only CL72:8 but also the total amount of CL, indicating that KMP could promote the CL synthesis pathway. This is consistent with the finding that KMP promotes LA uptake via *CPT1A*. Previous studies have shown that CL is required for maintaining CPT1 activity and ATP production [38,39]. Overall, the KMP-induced increase in the total amount of CL and the improvement in the quality of CL molecular species may have led to mitochondrial activation.

In all LA-treated groups, the levels of MLCL molecular species with acyl groups having more double bonds increased, whereas those of species with acyl groups with fewer double bonds decreased. Therefore, as CL matures owing to CL remodeling, the length of the acyl chain of MLCL increases, and MLCL also matures. The addition of KMP increased the number of MLCL molecular species with acyl groups having more double bonds, compared to the effect of adding LA alone, which could have occurred in conjunction with an increase in mature CL levels. Furthermore, the decreasing trend in the total amount of MLCL in the LA groups suggested that the decrease in MLCL synthesis was linked to a decrease in CL synthesis. Following KMP addition, this decrease was suppressed to a level comparable to that in the control group. These results suggest that KMP promoted the overall synthesis of MLCL, including MLCL molecular species with multiple double bonds.

Although the total MLCL/total CL ratio increased with the addition of LA alone, it decreased to the level observed in the control group with the addition of KMP. TAZ is necessary for CL remodeling [40,41,42]. Barth syndrome (BTHS), characterized by mutations in the *TAZ* gene, is associated with a defect in CL synthesis. MLCL accumulates in the mitochondria of patients with BTHS, resulting in an increased MLCL/CL ratio [43,44]. Consequently, energy metabolism is abnormal in patients with BTHS, resulting in a significant decline in movement function. Therefore, the effect of KMP in improving the MLCL/CL ratio in our fatty liver cell model suggests a potential role of KMP in promoting energy metabolism and contributing to the prevention and treatment of MASLD in the future.

## 5. Conclusions and Future Remarks

We demonstrated, using cultured liver cells, that KMP enhanced mitochondrial respiratory function, including ATP production, which is associated with the activation of β-oxidation based on the increase in the expression level of *CPT1A* and improvement in CL metabolism. Thus, the potential of KMP to reduce the MLCL/CL ratio in the fatty liver cellular model may foster energy metabolism, offering prospects for preventing and treating MASLD. In future studies, improving the balance of the MLCL/CL ratio stands out as a promising avenue for developing functional foods against mitochondrial dysfunction. Our study is limited in that it was conducted in a cell-based model, raising the possibility that the findings may not entirely reflect the results under physiological conditions. The use of another cell line could enhance the robustness of the evidence supporting the efficacy of KMP. Thus, in vivo experiments are needed to verify the effects of KMP on CL and energy metabolism on MASLD. The insights from our study using a fatty liver cell model are crucial in shaping future animal and human studies.

## Data Availability

Data are contained within the article and supplementary materials.

## Supplementary Materials

The following supporting information can be downloaded at: https://www.mdpi.com/article/10.3390/nu16040508/s1. Table S1: Experimental design in cell viability, mitochondrial function, and real-time PCR; Table S2: Experimental design in lipidomic analysis; Table S3: Primer sets used in this study; Figure S1: Cytotoxicity test in linoleic acid (LA)-loaded fatty liver cell model supplemented with or without kaempferol (KMP).
