# Supplementary material for: Kaempferol Improves Cardiolipin and ATP in Hepatic Cells: A Cellular Model Perspective in the Context of Metabolic Dysfunction-Associated Steatotic Liver Disease
Source: Nutrients. 2024 Feb 11;16(4):508. doi: 10.3390/nu16040508 (PMC10892986; doi:10.3390/nu16040508)
Supplement: Supplementary file 1 [file nutrients-16-00508-s001.zip › nutrients-2826783-supplementary.pdf]

Supplementary data

Table S1. Experimental design in cell viability, mitochondrial function, and real-time PCR.

	Cell viability		Mitochondrial function		real-time PCR	
Group	KMP (μM)	Incubation time (hr)	KMP (μM)	Incubation time (hr)	KMP (μM)	Incubation time (hr)
Control	0	24	0	24	0	6
KMP	0.001 – 100	24	10	24	1, 10	6

Table S2. Experimental design in lipidomic analysis.

	CL and MLCL		
Group	LA (mM)	KMP (μM)	Incubation time (hr)
Control	0	0	24
LA	0.8	0	24
LA + KMP1	0.8	1	24
LA + KMP10	0.8	10	24

Table S3. Primer sets used in this study.

Gene	Forward primer (5'-3')	Reverse primer (5'-3')
CPT1A	CTTTGGCCCTGTAGCAGATGA	TCGTCTCTGAGCTTGAGAACTT
SIRT3	TGGAAAGCCTAGTGGAGCTTCTGGG	TGGGGGCAGCCATCATCCTATTTGT
FOXO3A	TTCAAGGATAAGGGCGACAGCAAC	CTGCCAGGCCACTTGGAGAG
PPARGC1A	TGAACTGAGGGACAGTGATTTC	CCCAAGGGTAGCTCAGTTTATC
TFAM	ATAGGCACAGGAAACCAGTTAG	GCAGAAGTCCATGAGCTGAATA
ND1	ATGGCCAACCTCCTACTCCT	GCGGTGATGTAGAGGGTGAT
β-actin	CCAACCGCGAGAAGATGA	CCAGAGGCGTACAGGGATAG
GAPDH	GAAGGTGAAGGTCGGAGTC	GAAGATGGTGATGGGATTTC

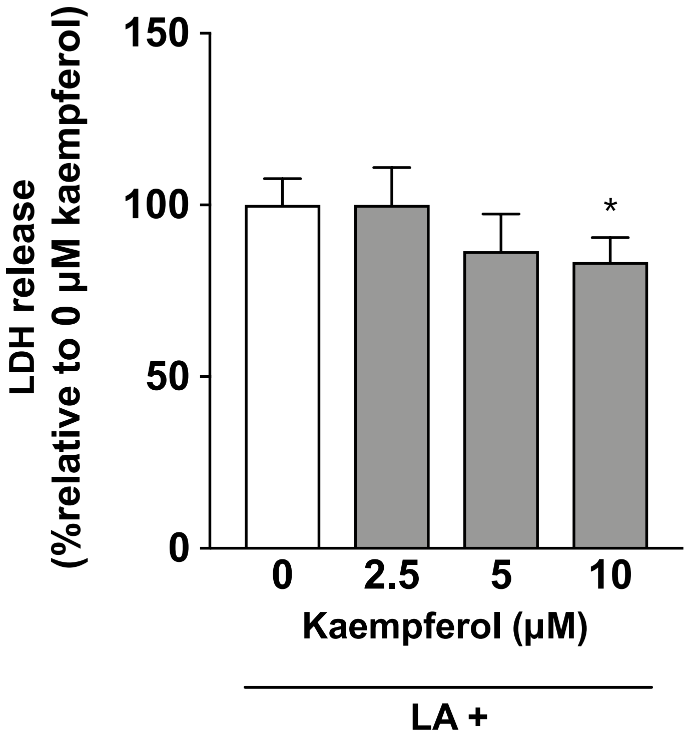

Figure S1. Cytotoxicity test in linoleic acid (LA)-loaded fatty liver cell model supplemented with or without kaempferol (KMP). Lactate dehydrogenase (LDH) in the supernatant following stimulation. * $P < 0.05$, one-way analysis of variance (ANOVA) with Dunnet's multiple comparisons test compared to the 0 μM KMP group ($n = 6$ for each group).
